# Blocking Notch signal in myeloid cells alleviates hepatic ischemia reperfusion injury by repressing the activation of NF-κB through CYLD

**DOI:** 10.1038/srep32226

**Published:** 2016-09-29

**Authors:** Heng-Chao Yu, Lu Bai, Zhao-Xu Yang, Hong-Yan Qin, Kai-Shan Tao, Hua Han, Ke-Feng Dou

**Affiliations:** 1 Department of Hepatic Surgery, Xi-Jing Hospital, Fourth Military Medical University, Xi’an, China; 2 Department of Clinical Laboratory, Xi-Jing Hospital, Fourth Military Medical University, Xi’an, China; 3 Department of Medical Genetics and Developmental Biology, Fourth Military Medical University, Xi’an 710032, China

## Abstract

Ischemia-reperfusion (I/R) is a major reason of hepatocyte injury during liver surgery and transplantation. Myeloid cells including macrophages and neutrophils play important roles in sustained tissue inflammation and damage, but the mechanisms regulating myeloid cells activity have been elusive. In this study, we investigate the role of Notch signaling in myeloid cells during hepatic I/R injury by using a mouse model of myeloid specific conditional knockout of RBP-J. Myeloid-specific RBP-J deletion alleviated hepatic I/R injury. RBP-J deletion in myeloid cells decreased hepatocytes apoptosis after hepatic I/R injury. Furthermore, myeloid-specific RBP-J deletion led to attenuated inflammation response in liver after I/R injury. Consistently, Notch blockade reduced the production of inflammatory cytokines by macrophages *in vitro*. We also found that blocking Notch signaling reduced NF-κB activation and increased cylindromatosis (CYLD) expression and knockdown of CYLD rescued reduction of inflammatory cytokines induced by Notch blockade in macrophages during I/R injury *in vitro*. On the other hand, activation of Notch signaling in macrophages led to increased inflammatory cytokine production and NF-κB activation and decreased CYLD expression *in vitro*. These data suggest that activation of Notch signaling in myeloid cells aggravates I/R injury, by enhancing the inflammation response by NF-κB through down regulation of CYLD.

Warm ischemia reperfusion (I/R) injury of liver takes place under a number of clinical conditions, including hepatic resection surgery, liver transplantation, and hemorrhagic shock with fluid resuscitation^1^. Liver I/R injury involves a continuous process of inflammation and immune responses including the generation of danger-associated molecular patterns (DAMPs), activation of endothelial cells, recruitment and activation of immune cell populations, increased expression and release of adhesion molecules and cytokines, and over production of free radicals, ultimately resulting in hepatocyte death^2^,^3^. Accumulating evidence has indicated that liver damage is triggered by accumulation of reactive oxygen species (ROS) generated in hepatocytes, macrophages and neutrophils in the early reperfusion phase which leads to necrosis and releasing DAMPs^4^. When activated by DAMPs, macrophages release a large quantity of pro-inflammatory cytokines, including TNF-α, IL-1β, among others, which are identified as contributing events to the inflammation-associated damage^5^,^6^,^7^.

Myeloid cells including neutrophils and macrophages have been considered as cell populations critically involved in liver I/R injury^8^,^9^,^10^. Established theory demonstrates that Kupffers’ cells are activated by oxidative stress in the initial phase of I/R injury and then activated macrophages produce massive ROS and pro-inflammatory cytokines which can recruit neutrophils to the site of I/R-induced inflammation. Depletion of neutrophil can protect mice from hepatic I/R injury. The critical role of neutrophils in I/R injury has been well established, and neutrophil inhibitors are considered promising treatments of I/R injury^11^,^12^. Macrophages in liver include liver-resident Kupffer cells (KCs) and inflammatory macrophages recruited from circulation^13^. Upon stimulation with oxidative stress during I/R injury, macrophages are activated and elicit their pro-inflammatory and cell-damaging roles by massively changing their gene expression profiles through the modulation of a wide range of transcriptional factors^14^,^15^. Nuclear factor-κB (NF-κB) transcriptional factor is the central transcription factor in response to ROS and TNF-α stimulations^16^. In macrophages, activated NF-κB mediates major inflammatory responses and exacerbates liver damage by up-regulation of various pro-inflammatory cytokines^6^,^17^. Therefore, a full understanding the regulation of NF-κB is critical to unveil the mechanism of liver I/R injury.

The Notch signaling pathway is highly conserved through evolution and regulates cell proliferation, apoptosis, and cell fate decisions in a broad range of tissues^18^. In mammals, four Notch receptors (Notch1-4) and five ligands (Jagged1, 2, and Delta-like [Dll] 1, 3, and 4) have been identified. Canonical Notch activation involves consecutive enzymatic receptor cleavages within the transmembrane domain executed by γ secretase-mediated reactions. This process releases Notch intracellular domain (NICD) that subsequently translocates into the nucleus, where it interacts with the transcription factor C promoter-binding factor 1/recombination signal binding protein Jκ (RBP-J). This protein-protein interaction leads to the dissociation of the RBP-J-centered transcription repression complex and the subsequent formation of a transcription activation complex, including Mastermind-like and p300/CBP, which transactivates the transcription of target genes such as the hairy and enhancer of split (Hes) family basic helix-loop-helix (bHLH) factors^19^,^20^. Our previous study have revealed that blockade of canonical Notch signaling by RBP-J deletion in hepatocytes increases ROS through JAK2/STAT3 signal and aggravates hepatocyte death in hepatic I/R injury^21^. As myeloid cells play a key role in the process of hepatic I/R injury, in this study, we investigate the role of Notch signal in myeloid cells in hepatic I/R injury by using myeloid-specific conditional knockout mice. Our results have demonstrated that blockade of Notch signaling by RBP-J deletion in myeloid cells alleviates hepatic I/R injury by compromised NF-κB activation through cylindromatosis (CYLD) up-regulation.

## Results

### Myeloid-specific RBP-J deletion alleviated hepatic I/R injury

**Figure 1:**
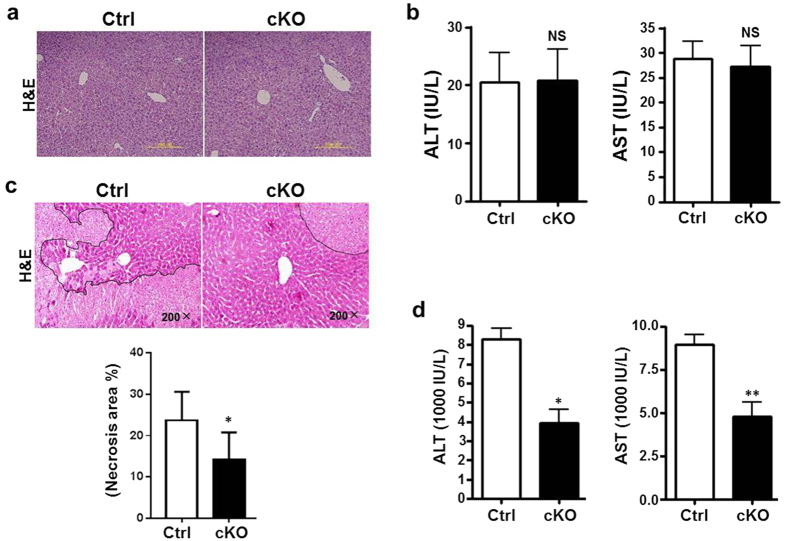
Myeloid-specific *RBP-J* deletion alleviated hepatic I/R injury *in vivo*. **(a,b**) Liver morphology and function in RBP-J cKO mice. Lyz2-Cre transgenic mice were crossed with RBP-J^f/+^ mice to obtain Lyz2-Cre-RBP-J^+/+^ (Ctrl) and Lyz2-Cre-RBP-J^f/f^ (cKO) mice. Liver sections were made from 6-weeks-old mice and stained by H&E staining (**a**). Serum ALT (left) and AST (right) were examined and compared between the cKO and control groups (**b**). **(c,d)** *RBP-J* cKO and control mice were subjected to hepatic ischemia, and examined 6 h post reperfusion. Liver sections were stained by H&E staining, with the outlined areas showing hepatic necrosis (c upper) and the necrosis area was quantified (c lower). Serum ALT (left) and AST (right) were determined (**d**). Bars = mean ± SD (n = 5). *P < 0.05, **P < 0.01, ns, not significant.

To investigate the role of Notch signaling in myeloid cells that are critically involved in hepatic I/R injury, we employed myeloid-specific conditional knockout mice of RBP-J, the key DNA-binding protein mediating signaling from all four mammalian Notch receptors. RBP-J-floxed mice were mated with Lyz2-Cre mice to obtain RBP^f/f^-Lyz2Cre (RBP-J cKO) mice, with RBP^+/+^-Lyz2Cre mice as a control. We first examined liver structure and function of the RBP-J cKO mice. Histological analysis of liver showed that there is no obvious difference between the RBP-J cKO mice and the control mice (Fig. 1a). Analysis of serum alanine aminotransferase (ALT) and aspartate aminotransferase (AST) indicated that mice with RBP-J cKO had the same level of ALT and AST as the control mice (Fig. 1b). Similar results were demonstrated in sham control (Supplementary Figure S1a,b). These results suggested that conditional RBP-J deletion in myeloid cells did not influence the gross structure and function liver in mice.

The RBP-J cKO mice were subjected to hepatic I/R injury. Histological examination of liver showed that I/R resulted in decreased tissue degeneration and focal necrosis in the RBP-J cKO mice as compared with the control mice 6 h post the reperfusion (Fig. 1c). Moreover, significantly lower level of serum ALT and AST was detected in the RBP-J cKO mice (Fig. 1d). These results suggested that deletion of RBP-J in myeloid cells alleviated liver damage induced by I/R injury.

### RBP-J deletion in myeloid cells decreased apoptosis after hepatic I/R injury

**Figure 2:**
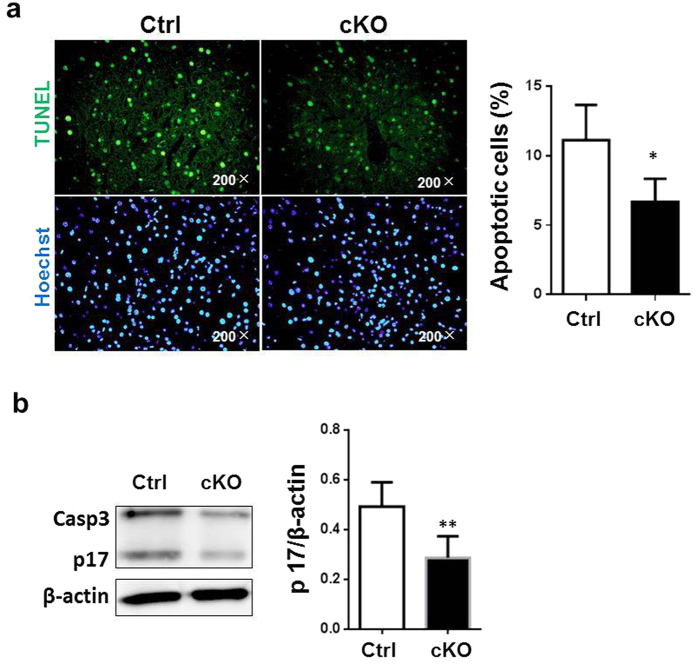
*RBP-J* disruption in myeloid cells led to decreased apoptosis after hepatic I/R injury. *RBP-J* cKO and control mice were subjected to hepatic ischemia, and analyzed 6 h post reperfusion. (**a**) Liver sections were stained with TUNEL (left), and the number of apoptotic cells was determined and compared quantitatively between the *RBP-J* cKO and control mice (right). (**b**) Total protein of liver was prepared. The protein level of cleaved caspase-3 p17 and caspase-3 were evaluated by using Western blot, with β-actin as a reference control (left). The bands were quantitatively compared between the *RBP-J* cKO and control mice (right). Bars = mean ± SD (n = 5). *P < 0.05, **P < 0.01.

We then determined apoptosis of hepatocytes in the RBP-J cKO and control mice after I/R injury. TUNEL staining detected significantly less apoptotic cells in liver sections of the RBP-J cKO mice 6 h after the reperfusion (Fig. 2a). Consistently, caspase-3 activity decreased in liver of the RBP-J cKO mice after I/R injury, as compared with the controls showed by cleaved caspase-3 p17 (Fig. 2b). These results indicated that disruption of Notch signaling in myeloid cells resulted in attenuated apoptosis of hepatocyte after I/R injury in mice.

### Myeloid-specific RBP-J deletion led to attenuated inflammation response in liver after I/R injury

**Figure 3:**
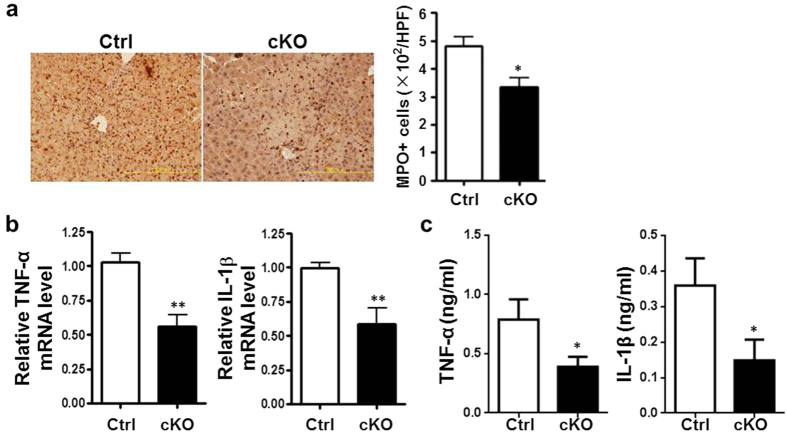
Myeloid-specific *RBP-J* deletion induced attenuated inflammation in liver after I/R injury. *RBP-J* cKO or control mice were subjected to hepatic ischemia, and analyzed 6 h post reperfusion. (**a**) Liver sections were immunostained with the anti-MPO antibody (left), and MPO-positive cells were quantitative compared between the *RBP-J* cKO and control mice (right). (**b**) Total RNA was extracted from liver tissue. The mRNA level of TNF-α (left) and IL-1β (right) was analyzed by using real-time RT-PCR, with β-actin as a reference control. (**c**) Serum TNF-α (left) and IL-1β (right) level was determined by using ELISA. Bars = mean ± SD (n = 5). *P < 0.05, **P < 0.01.

We next examined inflammatory response in liver of the RBP-J cKO and control mice after I/R injury by detecting neutrophil infiltration and the production of inflammatory cytokines in liver. The staining of myeloperoxidase (MPO) indicated that there was significantly reduced number of neutrophils infiltrating the liver of RBP-J cKO mice after reperfusion as compared with the control mice (Fig. 3a). The mRNA level of TNF-α and IL-1β decreased remarkably in the liver of RBP-J cKO mice after reperfusion, as compared with the control (Fig. 3b). Consistently, serum level of TNF-α and IL-1β was also reduced in the RBP-J cKO mice as compared with the control mice (Fig. 3c). These results demonstrated that myeloid-specific RBP-J deletion induced attenuated inflammation in liver after I/R injury.

### Notch blockade reduced the production of inflammatory cytokines by macrophages *in vitro*

**Figure 4:**
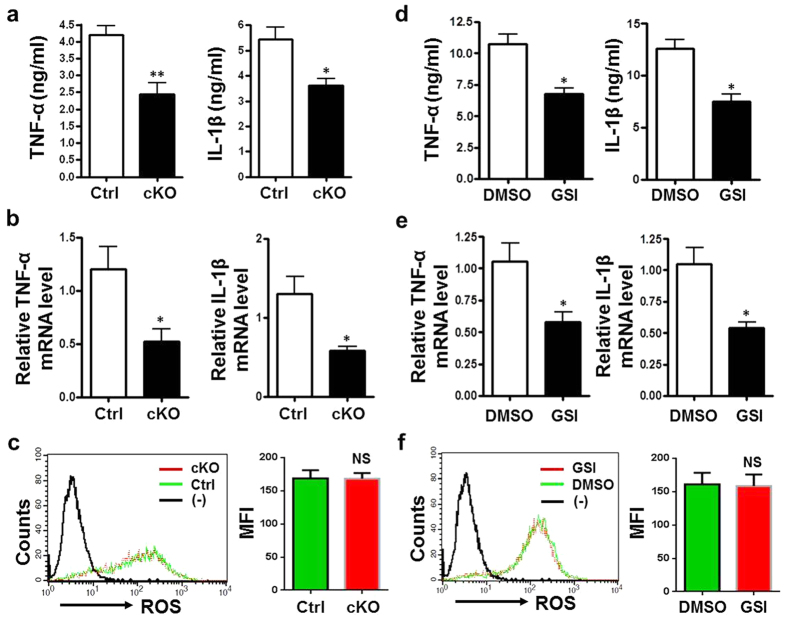
Myeloid-specific *RBP-J* deletion reduced the production of TNF-α and IL-1β by macrophages treated with conditional medium derived from I/R-injured hepatocytes *in vitro*. (**a–c**) BMDMs were generated from *RBP-J* cKO and control BM monocytes, and stimulated with CM prepared from Hepa1-6 cells that were subjected to I/R injury *in vitro*. The protein level of TNF-α and IL-1β in the supernatant was determined by using ELISA (**a**). Total RNA was extracted from the RBP-J cKO and control BMDMs stimulated with CM, and the mRNA level of TNF-α and IL-1β was examined by using real-time RT-PCR, with β-actin as a reference control (**b**). The level of ROS in the RBP-J cKO and control BMDMs was examined by using FACS, and was quantified with MFI (**c**). (**d–f**) RAW264.7 cells were treated with GSI or DMSO and stimulated simultaneously with CM prepared from Hepa1-6 cells that were subjected to I/R injury *in vitro*. The protein level of TNF-α and IL-1β in the supernatant was determined by using ELISA (**d**). Total RNA was extracted from the GSI- or DMSO-treated BMDMs stimulated with CM, and the mRNA level of TNF-α and IL-1β was examined by using real-time RT-PCR, with β-actin as a reference control (**e**). The level of ROS in the GSI- or DMSO-treated BMDMs was examined by using FACS, and was quantified with MFI (**f**). Bars = mean ± SD (n = 5). *P < 0.05, **P < 0.01, ns, not significant.

We then examined the response of macrophages with normal or interrupted Notch signaling to hepatocytes suffering from I/R injury. A hepatocyte line Hep1-6 was cultured and treated with I/R injury *in vitro*. The culture medium of the I/R-injured Hep1-6 cells was collected, and was used as a conditional medium (CM) (Supplementary Figure S2) to stimulate primary BM-derived macrophages (BMDMs) from the RBP-J cKO or control mice. Analysis of TNF-α and IL-1β in supernatants of BMDMs culture indicated that compared with the control, BMDMs from the RBP-J cKO mice produced less TNF-α and IL-1β (Fig. 4a). We also extracted RNA of BMDMs, and examined mRNA expression of TNF-α and IL-1β. The results showed that lower level of mRNA expression of TNF-α and IL-1β was detected in RBP-J cKO BMDMs than in the control BMDMs (Fig. 4b). The level of ROS in the BMDMs culture did not exhibit obvious difference between the RBP-J cKO and control groups (Fig. 4c).

We also tested the effect of CM of I/R-injured hepatocytes on mouse macrophage cell line RAW264.7 in the presence of GSI. The results showed that In RAW264.7 cells, blocking Notch signaling with GSI also led to decreased TNF-α and IL-1β production at both of protein and mRNA level (Fig. 4d,e). ROS level showed no significant difference between the GSI- and DMSO-treated macrophages (Fig. 4f). These results indicated that myeloid-specific Notch blockade reduced the production of inflammatory cytokines by macrophages *in vitro*.

### Decreased NF-κB activation and increased CYLD expression in macrophages with Notch blockade in I/R injury

**Figure 5:**
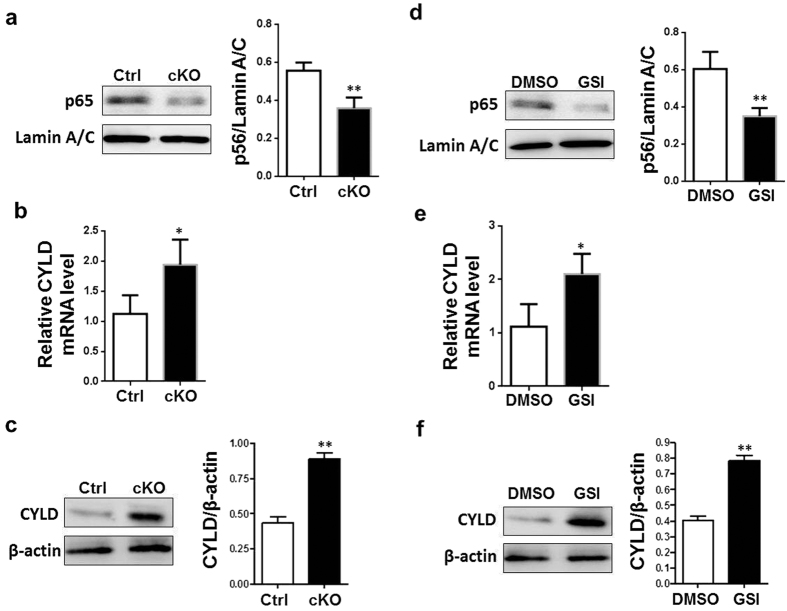
Blocking Notch signaling resulted in attenuated activation of NF-κB and up-regulation of CYLD. (**a–c**) BMDMs derived from the *RBP-J* cKO and control mice were stimulated with CM prepared from Hepa1-6 cell culture that had been treated with I/R injury *in vitro*. Nuclear proteins were extracted and p65 protein level was evaluated by using Western blot with LaminA/C as a reference control, and quantitatively compared between the RBP-J cKO and control group (**a**). Total RNA was extracted and the mRNA level of CYLD was analyzed by using real-time RT-PCR, with β-actin as a reference control (**b**). Total proteins were extracted and CYLD protein level was evaluated by using Western blot with β-actin as a reference control, and quantitatively compared between the RBP-J cKO and control group (**c**). **(d–f)** RAW264.7 cells were treated with GSI or DMSO and stimulated simultaneously with CM prepared from Hepa1-6 cells that were subjected to I/R injury *in vitro*. Nuclear proteins were extracted and p65 protein level was evaluated by using Western blot with LaminA/C as a reference control, and quantitatively compared (**d**). Total RNA was extracted and the mRNA level of CYLD was analyzed by using real-time RT-PCR, with β-actin as a reference control (**e**). Total proteins were extracted and CYLD protein level was evaluated by using Western blot with β-actin as a reference control, and quantitatively compared (**f)**. Bars = mean ± SD (n = 5). *P < 0.05, **P < 0.01.

We further examined the activation of NF-κB that is the central inflammatory transcription factor regulating the expression of TNF-α and IL-1β. *In vitro*, RBP-J cKO BMDMs stimulated by CM from I/R-injured hepatocytes showed decreased nuclear p65 level compared with the control, suggesting compromised NF-κB activation (Fig. 5a). Previous reports have demonstrated that Notch signal represses the expression of CYLD that negatively regulates NF-κB activation in macrophages^22^. We therefore determined mRNA and protein expression of CYLD in BMDMs treated with CM from I/R-injured hepatocytes. The results showed that blockade of Notch signal up-regulated expression of CYLD in BMDMs treated with CM from I/R-injured hepatocytes (Fig. 5b,c). Similar results were obtained with RAW264.7 cells treated with GSI or DMSO and CM from I/R-injured hepatocytes (Fig. 5d–f). *In vivo*, decreased nuclear p65 level and increased CYLD expression were observed in macrophages isolated from liver of RBP-J cKO mice compared to control mice (Supplementary Figure S3a–c).These results suggested that blocking Notch signaling reduced NF-κB activation in macrophages during I/R injury, likely through up-regulated CYLD expression.

### Knockdown of CYLD rescued compromised inflammatory response of macrophages induced by Notch blockade in I/R injury *in vitro*

**Figure 6:**
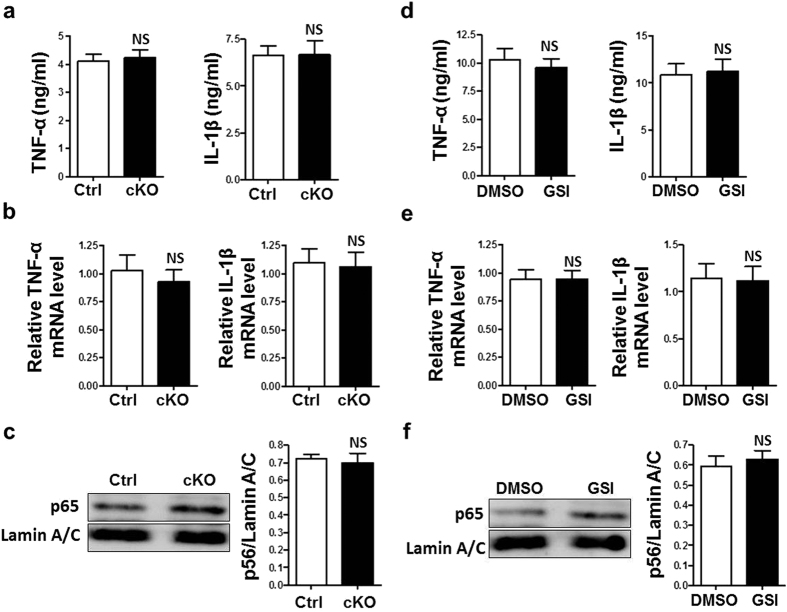
Knockdown of CYLD rescued decreased production of TNF-α and IL-1β and decreased activation of NF-κB induced by Notch blockade. (**a–c**) BMDMs generated from *RBP-J* cKO and control BM monocytes, with CYLD knockdown were stimulated with CM prepared from Hepa1-6 cells that were subjected to I/R injury *in vitro*. The protein level of TNF-α and IL-1β in the supernatant was determined by using ELISA (**a**). Total RNA was extracted and the mRNA level of TNF-α and IL-1β was examined by using real-time RT-PCR, with β-actin as a reference control (**b**). Nuclear proteins were extracted and p65 protein level was evaluated by using Western blot with LaminA/C as a reference control, and quantitatively compared between the RBP-J cKO and control group (**c**). (**d–f**) RAW264.7 cells were treated with GSI or DMSO with CYLD knockdown and stimulated simultaneously with CM prepared from Hepa1-6 cells that were subjected to I/R injury *in vitro*. The protein level of TNF-α and IL-1β in the supernatant was determined by using ELISA (**d**). Total RNA was extracted and the mRNA level of TNF-α and IL-1β was examined by using real-time RT-PCR, with β-actin as a reference control (**e**). Nuclear proteins were extracted and p65 protein level was evaluated by using Western blot with LaminA/C as a reference control, and quantitatively compared between the RBP-J cKO and control group (**f**). Bars = mean ± SD (n = 5). *P < 0.05, **P < 0.01, ns, not significant.

To further explore the role of increased CYLD in Notch blockade macrophages. The expression of CYLD was knocked down by using siRNA. RBP-J cKO BMDMs and control BMDMs were transfected with CYLD siRNA or SC RNA or NC RNA. The knockdown of CYLD was confirmed using qRT-PCR and western blot (Supplementary Figure S4a,b). These were also done with RAW264.7 macrophages treated with GSI or DMSO (Supplementary Figure S4c,d). Knockdown of CYLD rescued the expression of TNF-α and IL-1β at both the mRNA and protein levels in RBP-J cKO BMDMs (Fig. 6a,b). Consistently, Knockdown of CYLD rescued the decreased activation of NF-κB (Fig. 6c). Similar results were obtained with RAW264.7 cells treated with GSI or DMSO and CM from I/R-injured hepatocytes (Fig. 6d–f). These results suggested that increased CYLD were responsible for compromised inflammatory responses induced by Notch blockade.

### Activation of Notch signaling in macrophages led to increased inflammatory cytokine production and NF-κB activation *in vitro*

**Figure 7:**
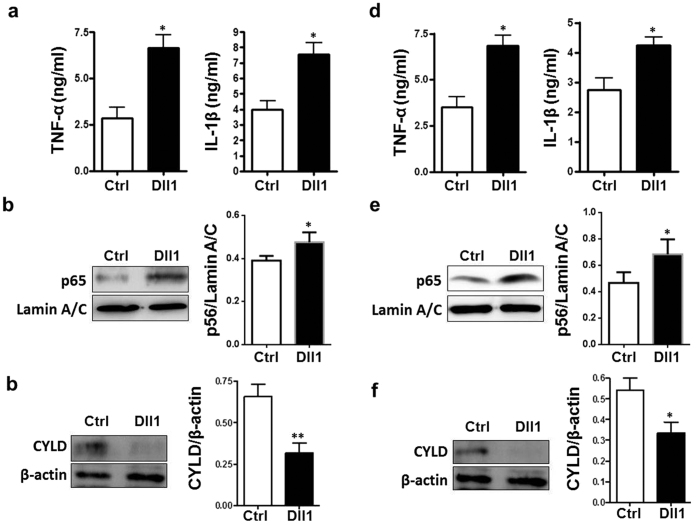
Forced Notch activation led to increased production of TNF-α and IL-1β and increased activation of NF-κB in macrophages suffering from I/R injury *in vitro*. (**a–c**) BMDMs were co-cultured with OP9-Dll1 (Dll1) or OP9-GFP (Ctrl) cells, and treated with CM prepared from Hepa1-6 cells that were subjected to I/R injury *in vitro*. The protein level of TNF-α and IL-1β in the supernatant was determined by using ELISA (**a**). Nuclear proteins were extracted and p65 protein level was evaluated by using Western blot with LaminA/C as a reference control, and quantitatively compared (**b**). Total proteins were extracted and CYLD protein level was evaluated by using Western blot with β-actin as a reference control, and quantitatively compared (**c**). (**d,e**) RAW264.7 cells were co-cultured with OP9-Dll1 or OP9-GFP cells, and were treated with CM prepared from Hepa1-6 cells that were subjected to I/R injury *in vitro*. The protein level of TNF-α and IL-1β in the supernatant was determined by using ELISA (**d**). Nuclear proteins were extracted and p65 protein level was evaluated by using Western blot with LaminA/C as a reference control, and quantitatively compared (**e**). Total proteins were extracted and CYLD protein level was evaluated by using Western blot with β-actin as a reference control, and quantitatively compared (**f**). Bars = mean ± SD (n = 5), *P < 0.05.

We next tested whether activation of Notch signaling augmented inflammatory response of macrophages using the macrophage culture system *in vitro*. BMDMs or RAW264.7 cells were co-cultured with OP9 cells over-expressing Dll1 and GFP or GFP only^23^, and were stimulated with CM from I/R-injured hepatocytes. Compared with cells co-cultured with OP9 over-expressing GFP, BMDMs and RAW264.7 co-cultured with OP9 over-expressing Dll1 and GFP expressed higher level of TNF-α and IL-1β production (Fig. 7a,d). Moreover, BMDMs and RAW264.7 cells were purified from the co-culture system by F4/80 staining followed by FACS sorting. Western blot analysis of nuclear extracts indicated that NF-κB activation was increased in both BMDMs and RAW264.7 cells with activated Notch signaling (Fig. 7b,e). Consistently, CYLD expression was decreased in BMDMs and RAW264.7 cells with activated Notch signaling (Fig. 7c,f). These results suggested that activation of Notch signaling in macrophages led to increased inflammatory cytokine production and NF-κB activation through down regulated CYLD expression during I/R injury.

## Discussion

Hepatic I/R injury is a pathophysiologic process initiated by the accumulation of ROS. Damaged parenchymal cells release DAMPs including HMGB1 and DNA/RNA, which are recognized by immune cells. And the infiltration and activation of innate immune cells especially neutrophils and macrophages are responsive for producing inflammatory cytokines/chemokines and augmented inflammatory response, leading to cell death and organ dysfunction. The critical role of neutrophils in I/R injury has been demonstrated, because depletion of neutrophil can protect mice from I/R injury^11^,^12^. Moreover, it has been shown that neutrophils are able to regulate the adaptive immune response through the secretion of IL-17, in liver and kidney I/R models^24^,^25^. Moreover, macrophages are believed to be the main source of inflammatory cytokines in hepatic I/R injury. Function of macrophages in I/R injury takes critical effect on the severity of I/R injury^26^. Macrophages depletion using liposomal clodronate led to less severe tubular necrosis mostly in a kidney I/R model. Depletion of CD11b positive cells including macrophages and monocytes by using transgenic mice conditionally expressing diphtheria toxin gene have shown that deficiency in these myeloid populations results in increased susceptibility to renal IR injury^27^.

However latest investigations have shown that myeloid cells exhibit extraordinary plasticity during inflammatory responses. When activated with different stimuli in different immune-microenvironment, macrophages are polarized into cells with essentially two distinct molecular phenotypes. The classically activated M1 macrophages express high level of inducible nitric oxide synthase (iNOS) and pro-inflammatory cytokines like TNF-α, IL-1β and IL-6. These macrophages initiate strong inflammation and are prone to aggravate tissue damage. On the other hand, the alternatively activated M2 macrophages express higher arginase-1 (Arg-1), mannose receptor (MR, CD206), and IL-10, and other molecules involved in anti-inflammation and tissue remodeling. This functionally plastic property may be involved in the function of macrophages during I/R injury^28^,^29^. Thus, it has suggested that macrophages may play a dual role in kidney I/R injury: at the early stage of kidney I/R injury, M1 macrophages secrete high levels of pro-inflammatory cytokines in renal tissues and contribute to the injury; at the late stage I/R injury, M2 macrophages contributed to tissue repair by suppressing the pro-inflammatory cytokine levels and secrete matrix components^30^. In the neutrophil compartment, similar cell subpopulations have also been identified, at least in tumors^31^. These findings raise the critical question of how the polarized activation of myeloid cells is achieved and regulated.

Previously we have shown in several inflammatory disease models that Notch signaling is critically involved in the regulation of macrophage polarization^22^,^32^. In the current study, we show for the first time that blockade of Notch signaling pathway in myeloid cells alleviated hepatic I/R injury. Our data indicated that deletion of the Notch signaling pathway led to decreased apoptosis and necrosis in liver, and caused attenuated inflammatory responses in hepatic I/R injury. It is important to notice that Lyz2-Cre-mediated RBP-J deletion happens in both macrophages and neutrophils. While literatures and the *in vitro* studies presented in the current study have shown that Notch disruption attenuated macrophage activation likely through CYLD-NF-κB, the exact role and mechanism of Notch signaling in neutrophils have not been directly accessed, although it could be speculated that Notch signal blockade reduced the pro-inflammatory activities of neutrophils.

By *in vitro* study, we show that Notch signaling regulates activation of NF-κB that is activated in inflammation and cell damage induced by I/R injury. Notch signal may interact with NF-κB pathway through regulating expression of CYLD in the hepatic I/R injury model. In previous studies we have demonstrated that Notch signaling protect hepatocytes from I/R injury by repressing ROS production through interaction with the JAK2/STAT3 pathway in hepatocytes^21^. However, in this study, we show that blockade of Notch signaling in myeloid cells alleviates hepatic I/R injury that is through regulation of NF-κB activation, accompanied by decreased production of inflammatory cytokines as TNF-α and IL-1β that are responsible for inflammation and tissue damage of liver. It seems likely that how Notch signaling works depends on the cell context. In different cell types it may interact with different signaling pathways and different molecules that lead to distinct effects. Mechanisms underlying different effects of the Notch signaling pathway on different types of cells need to be elucidated in the future.

These findings have potential translational implications. In hepatic I/R injury, Notch signaling effects on different populations of cells. If we activate it in hepatocytes, the ROS will be decreased that lead to alleviated apoptosis and necrosis. If we activate it in myeloid cells such as macrophages and probably also neutrophils, NF-κB activation will be increased that lead to more TNF-α and IL-1β production and more severe inflammation and tissue damage. So targeting Notch signaling in hepatic I/R injury should be cell type-specific.

## Methods

### Animals and treatment

Mice were maintained in a specific pathogen-free (SPF) condition on the C57BL/6 background. Mice carrying a Lyz2-Cre transgene (stock # 019096, Jackson Laboratory, Bar Harbor, ME) were crossed with RBP-J-floxed (RBP-J^f^) mice^33^, to obtain Lyz2-Cre transgenic with RBP-J^+/+^ (Control) or RBP-J^f/f^ (RBP-J cKO) mice. Mice were genotyped by using polymerase chain reaction (PCR) with tail DNA as a template. All experiments were approved by the Laboratory Animal Care and Use Committee of the Fourth Military Medical University and performed in accordance with a guideline from the Animal Experiment Administration Committee of the Fourth Military Medical University.

Hepatic I/R injury model induced by partial hepatic warm ischemia was established as described^21^. Briefly, mice were anesthetized by injection intraperitoneally (i.p) with sodium pentobarbital (60 mg/kg), and injected with heparin (100 U/kg). A midline laparotomy was performed, and an atraumatic clip was used to interrupt blood supply to the left lateral and median lobes of liver. After 90 min of hepatic ischemia, the clip was removed, initiating hepatic reperfusion. Mice were maintained on a heating pad (37 °C) to avoid temperature fall.

### Cell culture

For the isolation of hepatocytes and macrophages, mice were perfused with 15 ml of pre-warmed collagenase D (0.05%, Sigma-Aldrich) through the portal vein for 15 minutes. Livers were then removed and minced, and hepatocytes were pelleted by centrifugation at 50 g for 3 minutes three times. Single cell suspension in which hepatocytes were eliminated was prepared. Macrophages were purified by using successive gradient centrifugations on 8.2% Iodixanol (Optiprep, Axis-Shield). Hepatocytes were cultured as described^21^. BMDMs were cultured as described^32^. The mouse macrophage cell line RAW264.7 and mouse hepatocyte line Hepa1-6 were normally cultured with RPMI1640 medium supplemented with 10% fetal calf serum (FCS), 2 mM L-glutamine, 100 U/ml penicillin and 100 μg/ml streptomycin. For I/R injury of Hepa1-6 cells *in vitro*, Hepa1-6 cells were incubated in Krebs-Henseleit (KH) buffer, pH6.8, in a hypoxic chamber (0.5% O_2_) for 15 h. Subsequently, hypoxic KH was replaced by normaxic normal medium and were cultured further for 6 h^34^. The culture supernatants were collected and used as conditioned media (CM) to stimulate macrophages which was similar with CM from primary hepatocytes (Supplementary Figure S5). For transfection, cultured macrophages (5 × 10^5^) were seeded in 12-well plates, and transfected with 100 nmol/L CYLD small interfering RNA (siRNA) (5′-GGAAGAAGGUCGUGGUCAA-3′ and 5′-UUGACCACGACCUUCUUCC-3′) or scramble control siRNA (SC) (Qiagen, Germany) or control oligos (negative control [NC]) (Ribo bio, Guangzhou, China) by using Lipofectamine2000 (Invitrogen, Carlsbad, CA). Total RNA or proteins were prepared 48 hours after the transfection. In some cases, CM was included in the medium. The γ secretase inhibitor IX (GSI; Calbiochem, La Jolla, CA) was used at the concentration of 75 μM, with DMSO as a control. For co-culture of macrophages with OP9 stromal cells-derived cell lines, OP9-Dll1 or OP9-GFP cells^23^ (1 × 10^5^) were seeded in 12-well plates. After cell adherence, BMDMs or RAW264.7 cells (5 × 10^5^) were seeded and cultured further for 12 h and then stimulated with CM. The supernatants and cells were collected for further analysis.

## Additional Information

**How to cite this article**: Yu, H.-C. *et al*. Blocking Notch signal in myeloid cells alleviates hepatic ischemia reperfusion injury by repressing the activation of NF-κB through CYLD. *Sci. Rep.* **6**, 32226; doi: 10.1038/srep32226 (2016).

## Supplementary Material

Supplementary Information
