# Supplementary material for: Blocking Notch signal in myeloid cells alleviates hepatic ischemia reperfusion injury by repressing the activation of NF-κB through CYLD
Source: Sci Rep. 2016 Sep 29;6:32226. doi: 10.1038/srep32226 (PMC5041084; doi:10.1038/srep32226)
Supplement: Supplementary Information [file srep32226-s1.doc]

**Blocking Notch signal in myeloid cells alleviates hepatic ischemia reperfusion injury by repressing the activation of NF-κB through CYLD**

**Heng-Chao Yu^1†^, Lu Bai^2†^, Zhao-Xu Yang^1†^, Hong-Yan Qin^3^ , Kai-Shan Tao^1^, Hua Han^3*^, Ke-Feng Dou^1*^**

**Supporting methods**

**Histology.** Mice were sacrificed 6 h post reperfusion and the I/R-injured lobes were removed immediately and fixed in 10% formalin. Samples were embedded in paraffin and cut into 6 μm-thick sections. Samples were then stained with hematoxylin and eosin (H&E) staining, and images were taken under a microscope with a CCD camera.

Immunohistochemistry was performed by using standard procedures with rabbit anti-mouse myeloperoxidase (MPO) [^35^](#_ENREF_35) (Thermo, Astmoor Runcorn, UK) as a primary antibody, and the horseradish peroxidase-conjugated goat anti-rabbit immunoglobulin G (Boster BioTec, Wuhan, China) as a secondary antibody. Images were taken after development under a microscope with a CCD camera.

For the staining of apoptotic cells in tissue sections or cultured cells, a terminal deoxynucleotidyltransferase-mediated dUTP nick-end labeling (TUNEL) kit (Promega, Madison, WI) was employed according to the manufacturer’s protocol.

To quantify histological images, at least five random fields of each section were counted under microscope, and data were statistically compared between groups.

**Measurement of serum ALT and AST.** Mouse serum samples were collected at 6 h post reperfusion, and serum ALT and AST levels were determined by using a Chemistry Analyzer (AU400, Olympus, Tokyo, Japan).

**Flow cytometry.** For the sorting of macrophages from the co-culture with OP9 cells, the cells were trypsinized to prepared single cell suspensions. Cells were stained with PE-F4/80 (eBioscience) and were sorted by FACS for further analysis. For the measurement of intracellular ROS, BMDCs or RAW 264.7 cells were stained with 2’,7’-dichlorofluorescin (DCFH-DA) (Beyotime, Haimen, China) following the recommended protocols, and were analyzed by FACS. The level of intracellular ROS was quantified by using mean fluorescence intensity (MFI), and was statistically compared between groups.

**Enzyme-linked immunosorbent assay (ELISA).** Serum of mice or culture supernatants were collected and assayed for indicated cytokines by using ELISA kits (Xinshengbo Biotech, Shenzhen, China) following the recommended protocols.

**Western blot.** Cellular protein extracts were prepared from mouse liver or cultured cells with the Nuclear and Cytoplasmic Protein Extraction Kit (Beyotime, Haimen, China). Protein samples were subjected to SDS-polyacrylamide gel electrophoresis (PAGE), followed by electro-blotting onto PVDF membranes. The membranes were probed with primary antibodies including: anti-caspase3 (Santa Cruz), anti-cleaved caspase3 p17 (R&D Systems), anti-β-actin (Sigma-Aldrich), anti-p65, anti-CYLD (Cell Signaling), or anti-Lamin A/C (Sigma-Aldrich). As secondary antibodies, anti-rabbit-IgG (Boster Bio Tec) or anti-mouse-IgG (Boster Bio Tec) was used. Bands were revealed with enhanced chemiluminescence (ECL, Engreen, Beijing, China).

**Real-time reverse transcription (RT)-PCR.** Total RNA was prepared from tissues or cultured cells by using the Trizol reagent (Invitrogen, Carlsbad, CA) according to the manufacturer’s instructions. Complementary DNA was prepared using a reverse transcription kit (Takara, Otsu, Japan). Real-time PCR was performed by using a kit (SYBR Premix EX Taq, Takara, Otsu, Japan) and the ABI PRISM 7500 real-time PCR system, with β-actin as a reference control. Primers used in real-time PCR included: β-actin, 5’-CATCCGTAAAGACCTCTATGCCAAC and 5’-ATGGAGCCACCGATCCACA; Il-1β, 5’-TCCAGGATGAGGACATGAGCAC and 5’-GAACGTCACACACCAGCAGGTTA; TNF-α, 5’-CAGGAGGGAGAACAGAAACTCCA and 5’-CCTGGTTGGCTGCTTGCTT; Caspase 3, 5’-CTGCCGGAGTCTGACTGGAA and 5’-ATCAGTCCCACTGTCTGTCTCAATG; CYLD, 5'-GCCTGGCTTTTCTTTGACAG and 5'-GAAGGGCCATCATCAAAAGA.

**Statistics.** Statistical analysis was performed with the SPSS 12.0 program. Results were expressed as the means ± SD. The comparisons between groups were undertaken using the unpaired Student's t test. P < 0.05 was considered statistically significant.

**
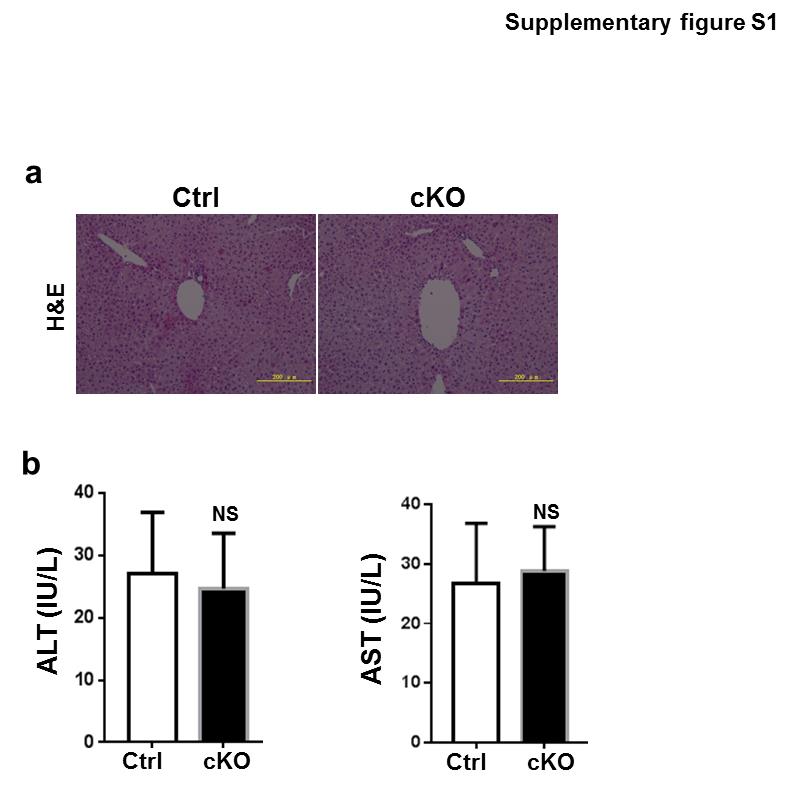
**

**Supplementary figure S1. Myeloid-specific *RBP-J* deletion led to no obvious change in sham control of hepatic I/R injury in vivo.** *RBP-J* cKO and control mice were subjected to sham surgery, and examined 6 h post-surgery. Liver sections were stained by H&E staining, (a). Serum ALT (left) and AST (right) were determined (b). Bars = mean ± SD (n = 5). ns, not significant.


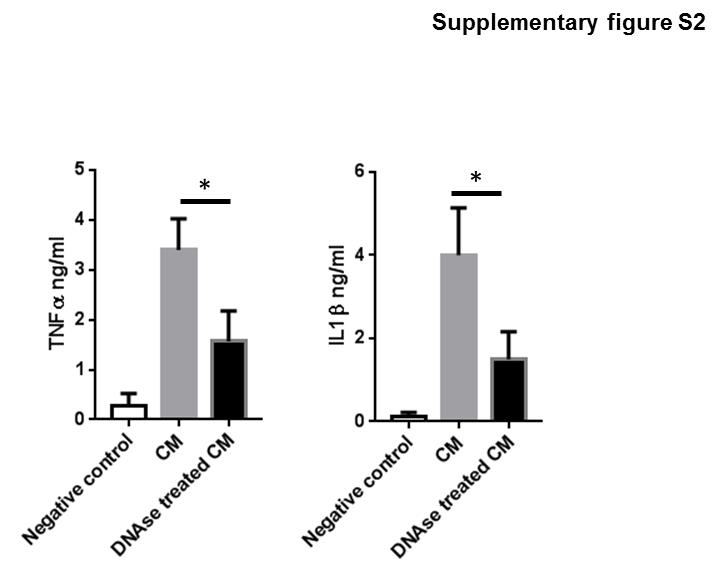


**Supplementary figure S2. DNAse reduced inflammatory cytokines production by RAW 264.7 cells stimulated by conditioned medium.** Conditioned medium were collected from I/R injured Hepa1-6 cells and were treated by DNAse or not 2 hours before being used to stimulate RAW264.7 cells. TNFα and IL1β were detected by ELISA. Bars = mean ± SD (n = 3). *, P < 0.05.

**
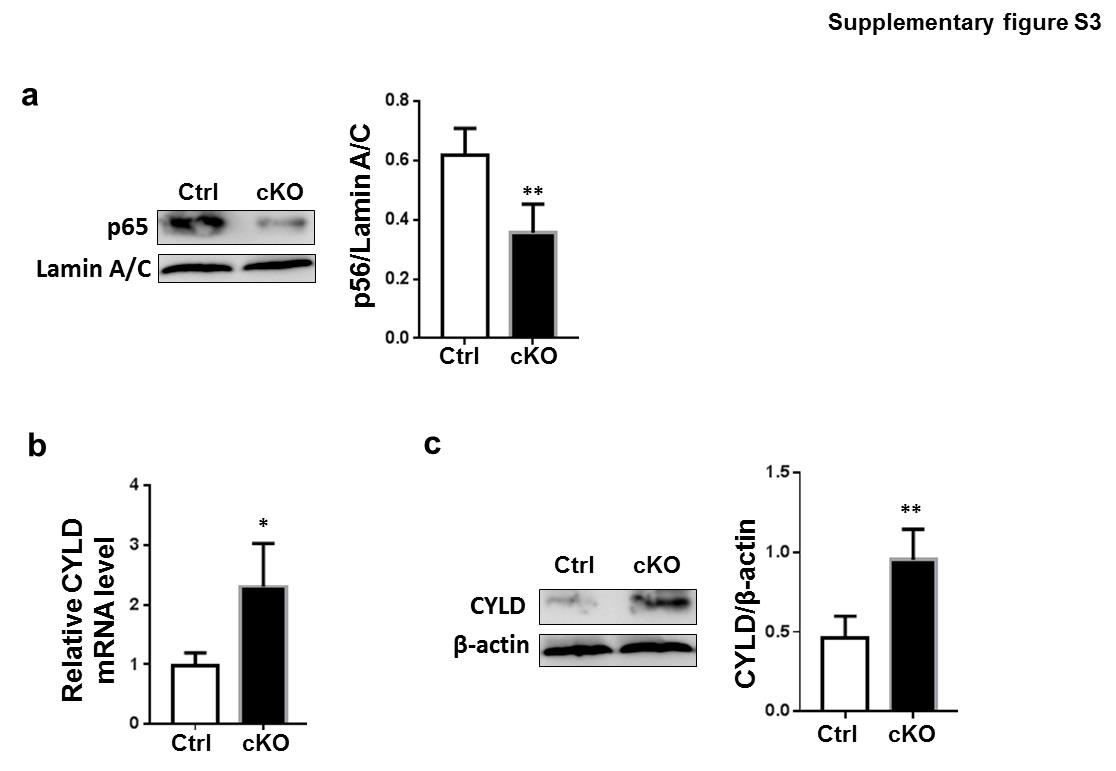
**

**Supplementary figure S3. Blocking Notch signaling resulted in attenuated activation of NF-κB and up-regulation of CYLD in macrophages in hepatic I/R injury. M**acrophages were isolated from liver of the *RBP-J* cKO and control mice subjected to hepatic I/R injury. Nuclear proteins were extracted and p65 protein level was evaluated by using Western blot with LaminA/C as a reference control, and quantitatively compared between the RBP-J cKO and control group (a). Total RNA was extracted and the mRNA level of CYLD was analyzed by using real-time RT-PCR, with β-actin as a reference control (b). Total proteins were extracted and CYLD protein level was evaluated by using Western blot with β-actin as a reference control, and quantitatively compared between the RBP-J cKO and control group (c). Bars = mean ± SD (n = 5). *, *P* < 0.05, **, *P* < 0.01.

**
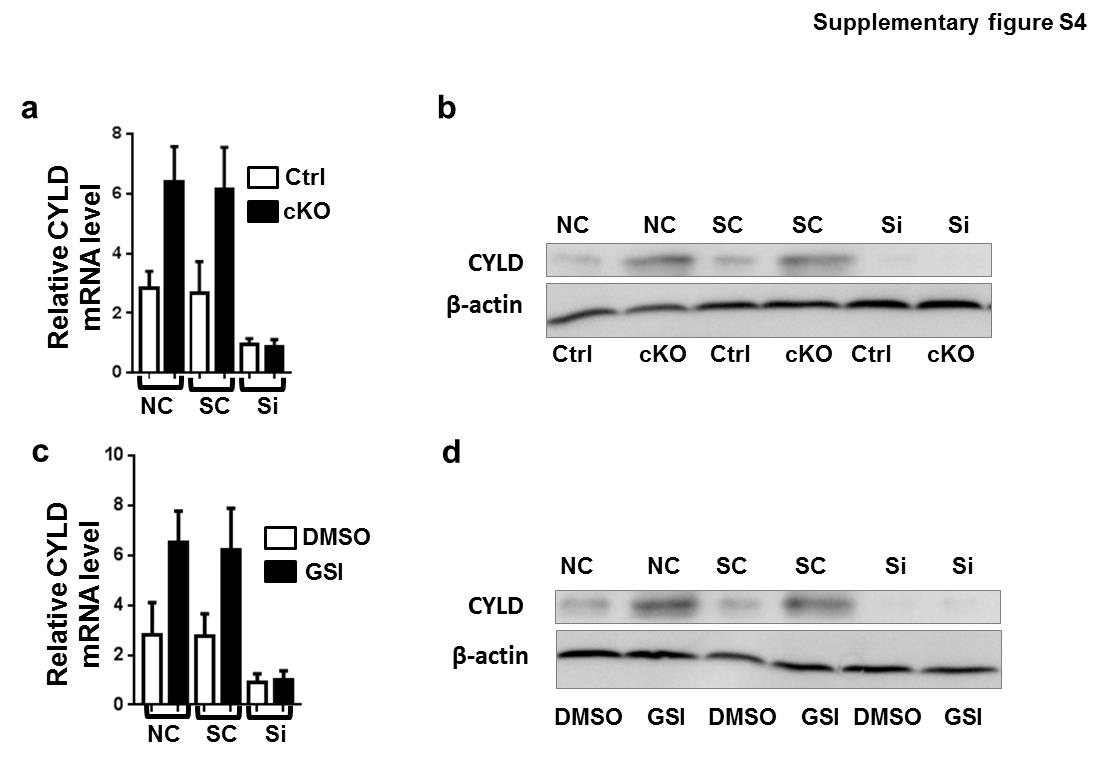
**

**Supplementary figure S4. Expression of CYLD was knocked down by transfected CYLD siRNA.** (a, b) BMDMs derived from the RBP-J cKO and control mice were transfected with CYLD siRNA or SC or NC. Total mRAN were extracted and the mRNA level of CYLD was examined by using real-time RT-PCR, with β-actin as a reference control (a). Total proteins were extracted and CYLD protein level was evaluated by using Western blot with β-actin as a reference control (b). (c, d) RAW264.7 cells were treated with GSI or DMSO were transfected with CYLD siRNA or NC. Total mRAN were extracted and the mRNA level of CYLD was examined by using real-time RT-PCR, with β-actin as a reference control (c). Total proteins were extracted and CYLD protein level was evaluated by using Western blot with β-actin as a reference control (d).


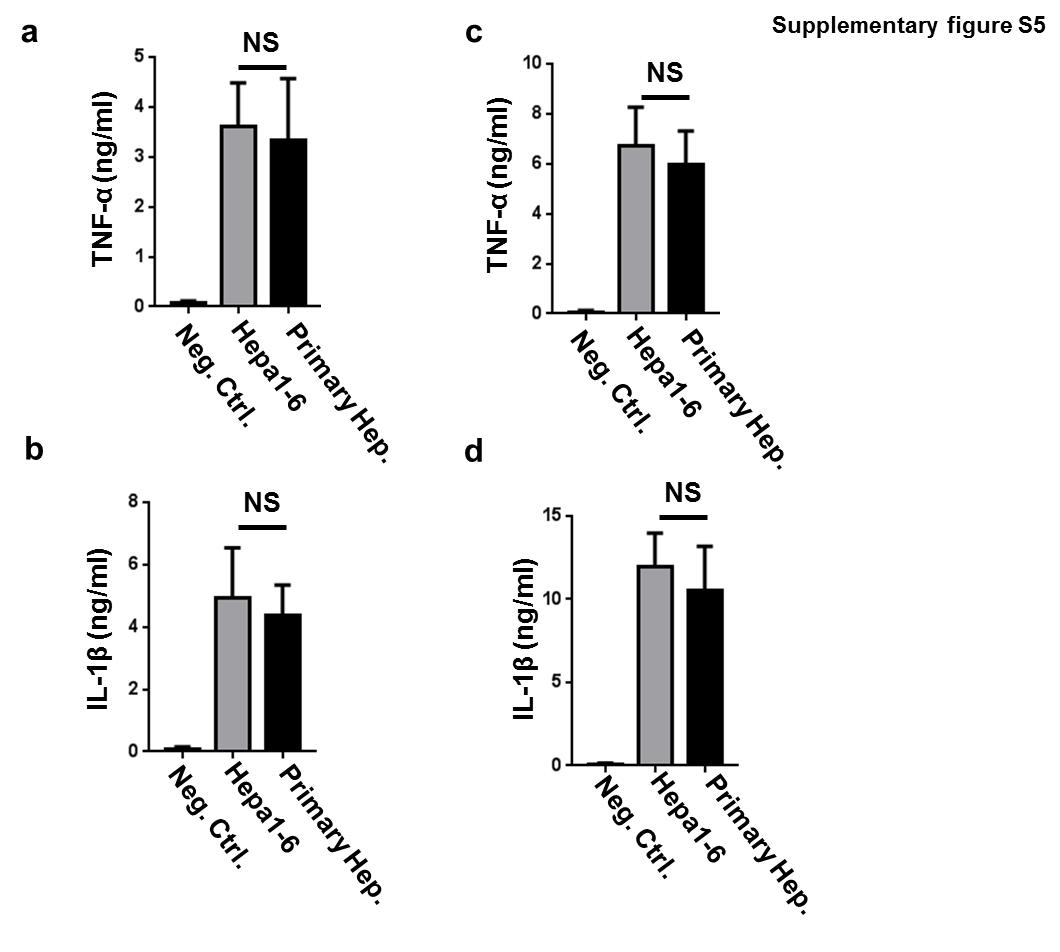


**Supplementary figure S5.** **Similar conditional medium can be derived from Hepa1-6 cells and primary hepatocytes.** (a, b) Hepa1-6 cells or primary hepatocytes were subjected to I/R injury in vitro and the supernatants were collected and used as conditional medium to stimulate macrophages from bone marrow. Production of TNF-α (a) and IL-1β (b) were detected by ELISA. (c, d) the Hepa1-6 cells or primary hepatocytes were subjected to I/R injury in vitro and the supernatants were collected and used as conditional medium to stimulate RAW264.7 cells. Production of TNF-α (a) and IL-1β (b) were detected by ELISA. Bars = mean ± SD (n = 5). NS, not significant.
